# A study of glycemic perturbations following two doses of COVID-19 vaccination for patients with diabetes: the impacts of vaccine type and anti-diabetes drugs

**DOI:** 10.1186/s13098-023-01059-0

**Published:** 2023-04-25

**Authors:** Cheng-Wei Lin, Shih-Yuan Hung, I-Wen Chen

**Affiliations:** Division of Endocrinology and Metabolism, Chang Gung Memorial Hospital at Linkou, 5, Fusing St., Guishan Dist, Taoyuan City, 333 Taiwan

**Keywords:** COVID-19, COVID-19 vaccine, Diabetes, Hyperglycemia, Sodium-glucose co-transporter 2 inhibitor (SGLT2i)

## Abstract

**Background:**

Glycemic monitoring has become critical during the COVID-19 pandemic because of poor prognosis in diabetes. Vaccines were key in reducing the spread of infection and disease severity but data were lacking on effects on blood sugar levels. The aim of the current study was to investigate the impact of COVID-19 vaccination on glycemic control.

**Methods:**

We performed a retrospective study of 455 consecutive patients with diabetes who completed two doses of COVID-19 vaccination and attended a single medical center. Laboratory measurements of metabolic values were assessed before and after vaccination, while the type of vaccine and administrated anti-diabetes drugs were analyzed to find independent risks associated with elevated glycemic levels.

**Results:**

One hundred and fifty-nine subjects received ChAdOx1 (ChAd) vaccines, 229 received Moderna vaccines, and 67 received Pfizer–BioNtech (BNT) vaccines. The average HbA1c was raised in the BNT group from 7.09 to 7.34% (*P* = 0.012) and non-significantly raised in ChAd (7.13 to 7.18%, *P* = 0.279) and Moderna (7.19 to 7.27%, *P* = 0.196) groups. Both Moderna and BNT groups had around 60% of patients with elevated HbA1c following two doses of COVID-19 vaccination, and the ChAd group had only 49%. Under logistic regression modeling, the Moderna vaccine was found to independently predict the elevation of HbA1c (Odds ratio 1.737, 95% Confidence interval 1.12–2.693, *P* = 0.014), and sodium-glucose co-transporter 2 inhibitor (SGLT2i) was negatively associated with elevated HbA1c (OR 0.535, 95% CI 0.309–0.927, *P* = 0.026).

**Conclusions:**

Patients with diabetes might have mild glycemic perturbations following two doses of COVID-19 vaccines, particularly with mRNA vaccines. SGLT2i showed some protective effect on glycemic stability. Hesitancy in having vaccinations should not be indicated for diabetic patients with respect to manageable glycemic change.

**Trial registration:**

Not applicable.

## Introduction

Under the health-threatening ongoing coronavirus disease 2019 (COVID-19) pandemic caused by severe acute respiratory syndrome coronavirus 2 (SARS-CoV-2), vaccines have become key in minimizing the spread of infection. Several vaccines for SARS-CoV-2 have gained emergency use authorization listing by the World Health Organization, with some being adenoviral vector vaccines (Oxford–AstraZeneca, and Janssen) and some being mRNA vaccines (Moderna, and Pfizer–BioNtech). Under the guidance of the Center for Disease Control, the general population has gradually received vaccinations since the local outbreak of COVID-19 in Taiwan in 2021.

Diabetes Mellitus is a chronic disease with high prevalence worldwide across ethnic groups, and those with diabetes appeared susceptible to COVID-19, receiving worse clinical outcomes [1, 2]. Accordingly, experts strongly advised patients with diabetes to be vaccinated against COVID-19; however, vaccine hesitancy existed among people even during the COVID-19 pandemic [3], and this barrier was also noted in patients with diabetes [4]. Such hesitancy was often attributed to unawareness of the risk of COVID-19, doubt in vaccine efficacy, and fear of the side effects [4]. General reported side effects were soreness, fatigue, myalgia, headache, chills, fever, joint pain and nausea, etc. [5].

Furthermore, worry was also driven by concerns about the temporary instability of blood glucose levels post-vaccination among diabetics. Temporal hyperglycemia was noted in one study conducted on changes in clinical laboratory measurements after COVID-19 vaccination [6], while several case reports also presented acute hyperglycemic emergencies after vaccination against COVID-19 [7–10]. However, inconsistent results were noted in continuous glucose monitoring for patients with type 1 diabetes post-vaccination [11, 12]. Based on these conflicting results, we decided to perform a study to assess the metabolic changes before and after COVID-19 vaccination among patients with diabetes, particularly focusing on glycemic control, with the additional aims of better characterizing the potential safety issues in glucose stability between different types of vaccines in the real world as well as focusing on the impact of anti-diabetes drugs.

## Materials and methods

### Participants

Four hundred and fifty-five consecutive patients with diabetes who were undergoing treatment in the Endocrinology Outpatient Department of Chang Gung Memorial Hospital between January 2021 and April 2022 were recruited. All the participants completed two doses of COVID-19 vaccines during the study observation period. The types of vaccines included ChAdOx1 nCov-19 (Oxford–AstraZeneca; hereafter referred to as ChAd), mRNA1273 (Moderna) and BNT162b2 (Pfizer–BioNtech, hereafter referred to as BNT) and the interval between vaccine doses ranged from 1 to 3 months. All of the participants underwent regular follow-ups and were treated by stable anti-diabetes regimens without changing doses during the enrolled time, and none were pregnant. No subject had an event of predisposing risks of hyperglycemia emergence neither in severe illness or surgery, nor abnormal fasting behavior, and they also had good adherence to medications throughout the study period. Additionally, no subject was exposed to COVID-19 infection or suffered from a vaccine-related severe adverse event such as anaphylaxis, thrombocytopenia, thromboembolism, myocarditis or Guillain-Barré syndrome, etc. The Institutional Review Board of Chang Gung Memorial Hospital approved this retrospective study (No. 202202371B0), while the reporting of this study conformed to STROBE guidelines [13].

### Clinical characteristics and data

The clinical characteristics of patients, including age, gender, diabetes duration, diabetes type and smoking or alcohol drinking habits as well as comorbidities including retinopathy, proteinuria, hypertension, coronary heart disease, cerebrovascular accident, heart failure and end-stage renal disease were recorded beginning at their clinic visit before the first dose of COVID-19 vaccination. Laboratory measurements to assess glycemic control, circulating lipid profile, circulating liver enzyme activities and renal function were made at the initial enrollment day within 90 days (median time: 43 days [first quartile 20, third quartile 64]) before the first dose of vaccine, and the end day within 90 days (median time: 42 days [20, 64]) after the second dose of vaccine. The anti-diabetes drugs for these patients during this treatment period included sulfonylurea, metformin, acarbose, glinide, pioglitazone, dipeptidyl peptidase-4 inhibitor (DPP4i), sodium-glucose co-transporter 2 inhibitor (SGLT2i), glucagon-like peptide 1 (GLP-1) analog and insulin.

### Statistical analysis

Comparisons between the three groups of different COVID-19 vaccines were performed using Pearson’s chi-square test for categorical variables or the one-way analysis of variance (one-way ANOVA) for continuous variables, as indicated. Paired sample *t*-tests were used to compare the biochemistry changes across the two doses of COVID-19 vaccination period. Three kinds of COVID-19 vaccines and factors of drug items under adjustment of clinical characteristics (age, gender, and comorbidities including retinopathy, proteinuria, hypertension, coronary heart disease, cerebrovascular accident, heart failure and end-stage renal disease) were entered into a multivariate logistic regression model to identify independent risk factors in predicting elevated HbA1c following vaccination. The odds ratios and 95% confidence interval of COVID-vaccines and anti-diabetes drugs correlated with elevated HbA1c are presented by forest plot in Fig. 2. All statistical analyses were performed using the Statistical Package for the Social Sciences (SPSS for Windows, version 19.0, Armonk, NY: IBM Corp.) software.

## Results

### Characteristics of patients receiving COVID-19 vaccines

Among the total participants, 159 subjects received ChAd vaccines, 229 received Moderna vaccines, and 67 received BNT vaccines. The comparison of clinical characteristics of the participants between receiving each kind of COVID-19 vaccines is shown in Table 1. The ages of these groups of participants differed, the youngest group being those patients receiving BNT vaccines with mean age of 52.62 years and mean diabetes duration of 7.39 years followed by the ChAd group (mean age 63.44 and diabetes duration 9.17) and then the Moderna group (mean age 65.67 and diabetes duration 10.57). The *post hoc* test (by Bonferroni) indicated the subjects with ChAd and Moderna vaccines had similar ages (*P* = 0.174) and differed from those receiving BNT vaccines (*P* < 0.001). Only five subjects had type 1 diabetes with one person in the ChAd group and two persons in both Moderna and BNT groups. According to the National Health Insurance Research Database, type 1 diabetes accounts for less than 0.6% of the entire diabetic population in our country, explaining the small number of cases in the current study [14]. Subtle male gender predominance was noted in all groups (56.33–68.66%, *P* = 0.201), with the proportions of diabetic complications being similar in retinopathy (17.91–21.83%, *P* = 0.782) and proteinuria (34.33–37.99%, *P* = 0.806) of all three groups. The proportions of associated comorbidities were highest in hypertension of all three groups, being 71.64–73.36% (*P* = 0.922) followed by coronary heart disease (8.73–11.94%, *P* = 0.709) and cerebrovascular accident (4.48–8.81%, *P* = 0.418), whereas heart failure and end-stage renal disease were seldom and predominantly present in subjects with ChAd vaccines (5.03% and 5.03% respectively).

**Table 1:** Clinical characteristics and anti-diabetes drugs of patients with diabetes receiving COVID-19 vaccines

Characteristic	Mean (standard deviation) or No. (%)						
	ChAd (n = 159)		Moderna (n = 229)		BNT (n = 67)		*P* value
Age (years)	63.44	(14.52)	65.67	(8.42)	52.62	(11.34)	< 0.001^*^
Male gender	89	(56.33%)	132	(57.64%)	46	(68.66%)	0.201
Diabetes duration (years)	9.17	(5.98)	10.57	(6.17)	7.39	(5.5)	< 0.001^*^
Type 1 Diabetes	1	(0.63%)	2	(0.87%)	2	(2.99%)	0.269
Alcohol	22	(13.84%)	40	(17.47%)	7	(17.47%)	0.313
Smoker	19	(11.95%)	35	(15.28%)	14	(20.90%)	0.222
Retinopathy	34	(21.38%)	50	(21.83%)	12	(17.91%)	0.782
Proteinuria	56	(35.44%)	87	(37.99%)	23	(34.33%)	0.806
Hypertension	114	(71.70%)	168	(73.36%)	48	(71.64%)	0.922
Coronary heart disease^a^	14	(8.81%)	20	(8.73%)	8	(11.94%)	0.709
Cerebrovascular accident^b^	14	(8.81%)	14	(6.11%)	3	(4.48%)	0.418
Heart failure	8	(5.03%)	4	(1.75%)	2	(2.99%)	0.183
End-stage renal disease	8	(5.03%)	1	(0.44%)	0	(0.00%)	0.003^*^
Anti-Diabetes drugs							
Sulfonylurea	54	(33.96%)	98	(42.79%)	28	(41.79%)	0.119
Metformin	133	(83.65%)	195	(85.15%)	59	(88.06%)	0.696
Acarbose	8	(5.03%)	8	(3.49%)	4	(5.97%)	0.609
Glinide	7	(4.40%)	7	(3.06%)	0	(0.00%)	0.216
Pioglitazone	5	(3.14%)	6	(2.62%)	1	(1.49%)	0.778
DPP4i^c^	65	(40.88%)	98	(42.79%)	25	(37.31%)	0.718
SGLT2i^d^	28	(17.72%)	34	(14.91%)	21	(31.82%)	0.007^*^
Insulin	30	(18.87%)	56	(24.45%)	15	(22.39%)	0.428
GLP-1^e^ analog	6	(3.77%)	11	(4.80%)	3	(4.48%)	0.888
Statins	112	(70.44%)	159	(69.43)	48	(71.64)	0.935

^*^ Significance: *P* value < 0.05

^a^ Coronary heart disease including history of ischemic heart disease or coronary artery disease

^b^ Cerebrovascular accident including history of embolic, ischemic, or hemorrhagic stroke

^c^ DPP4i = dipeptidyl peptidase 4 inhibitor

^d^ SGLT2i = sodium-glucose co-transporter 2 inhibitor

^e^ GLP-1 = glucagon-like peptide 1

### Medications

The dosage regimens of anti-diabetes drugs throughout the study period were not changed and are displayed in Table 1. The proportions of anti-diabetes drugs were similar between the three groups except for SGLT2i. Among these, around 34 ~ 43% of patients took sulfonylurea, 84 ~ 88% took metformin, 37 ~ 43% took DPP4i, and 19 ~ 24% underwent insulin injection therapy. SGLT2i was used by 17.72% of patients in the ChAd group, 14.91% in the Moderna group, and 31.82% in the BNT group (*P* = 0.007). The other anti-diabetes drugs including acarbose, glinide, pioglitazone, and GLP-1 analog were used by ≤ 10% of patients among the three groups without significant difference. Additionally, around 70% of patients in each group were taking statin as a treatment for hyperlipidemia (*P* = 0.935).

### Biochemistry changes

Table 2 expresses the serum biochemistry change. After the two doses of COVID-19 vaccination, all the participants had stable pre-meal (AC) sugar in each group of ChAd (from 127.81 to 131.29 mg/dL, *P* = 0.15), Moderna (from 132.34 to 131.02 mg/dL, *P* = 0.6), and BNT (from 130 to 133.78 mg/dL, *P* = 0.323). Nevertheless, the average serum glucose level had slightly but statistically significant increase in the BNT group with elevated HbA1c from 7.09 to 7.34% (*P* = 0.012), with non-significant increase in ChAd (7.13 to 7.18%, *P* = 0.279) and Moderna (7.19 to 7.27%, *P* = 0.196) groups. Regarding renal function, the estimated glomerular filtration rate (eGFR) was raised in groups of Moderna (76.79 to 79.97 ml/min/1.73m2, *P* = 0.001) and BNT (89.23 to 93.24 ml/min/1.73m2, *P* = 0.03), but stable in subjects with ChAd. The liver enzyme activity of alanine aminotransferase (ALT) was stable in all participants of three groups. Concerning lipid profile, subjects in the Moderna group had obvious lipid change with elevated cholesterol (160.46 to 164.98 mg/dL, *P* = 0.006), high-density lipoprotein (47.76 to 49.03 mg/dL, *P* = 0.004), low-density lipoprotein (89.18 to 93.09 mg/dL, *P* = 0.005) and downward triglyceride (158.48 to 138.15 mg/dL, *P* = 0.011).

**Table 2:** Biochemical parameters before and after two doses of the variant COVID-19 vaccines

	ChAd/ ChAd					Moderna/ Moderna					BNT/BNT				
	Before		After		*P*	Before		After		*P*	Before		After		*P*
AC sugar (mg/dL)	127.81	(32.04)	131.29	(39.76)	0.150	132.34	(40.31)	131.02	(31.15)	0.600	130.00	(38.48)	133.78	(34.77)	0.323
HbA1c (%)	7.13	(1.24)	7.18	(1.30)	0.279	7.19	(1.28)	7.27	(1.15)	0.196	7.09	(0.95)	7.34	(1.08)	0.012^*^
eGFR (ml/min/1.73m2) ^a^	83.96	(31.11)	84.44	(32.78)	0.712	76.79	(29.94)	79.97	(32.52)	0.001^*^	89.23	(27.53)	93.24	(32.55)	0.030^*^
ALT (U/L)	27.73	(19.11)	29.39	(25.78)	0.227	28.92	(20.69)	29.00	(20.03)	0.943	34.21	(28.76)	38.45	(33.62)	0.271
Cholesterol (mg/dL)	159.61	(30.50)	163.14	(29.95)	0.137	160.46	(28.00)	164.98	(30.81)	0.006^*^	170.39	(36.76)	177.16	(35.99)	0.108
Triglyceride (mg/dL)	143.63	(83.23)	141.70	(74.29)	0.699	158.48	(167.68)	138.15	(79.05)	0.011^*^	202.55	(281.03)	179.60	(133.49)	0.318
HDL (mg/dL)	47.02	(11.35)	48.00	(13.37)	0.076	47.76	(13.81)	49.03	(14.83)	0.004^*^	44.46	(9.55)	46.15	(9.90)	0.009^*^
LDL (mg/dL)	90.12	(26.85)	92.69	(24.36)	0.169	89.18	(23.29)	93.09	(27.87)	0.005^*^	98.87	(28.67)	104.49	(32.34)	0.055

^*^ Significance: *P* value < 0.05

^a^ Exclude subjects with end-stage renal disease

Note: AC = pre-meal, HbA1c = glycated hemoglobin, ALT = Alanine aminotransferase, eGFR = estimated glomerular filtration rate, HDL = high density lipoprotein, LDL = low density lipoprotein

**Fig. 1:**
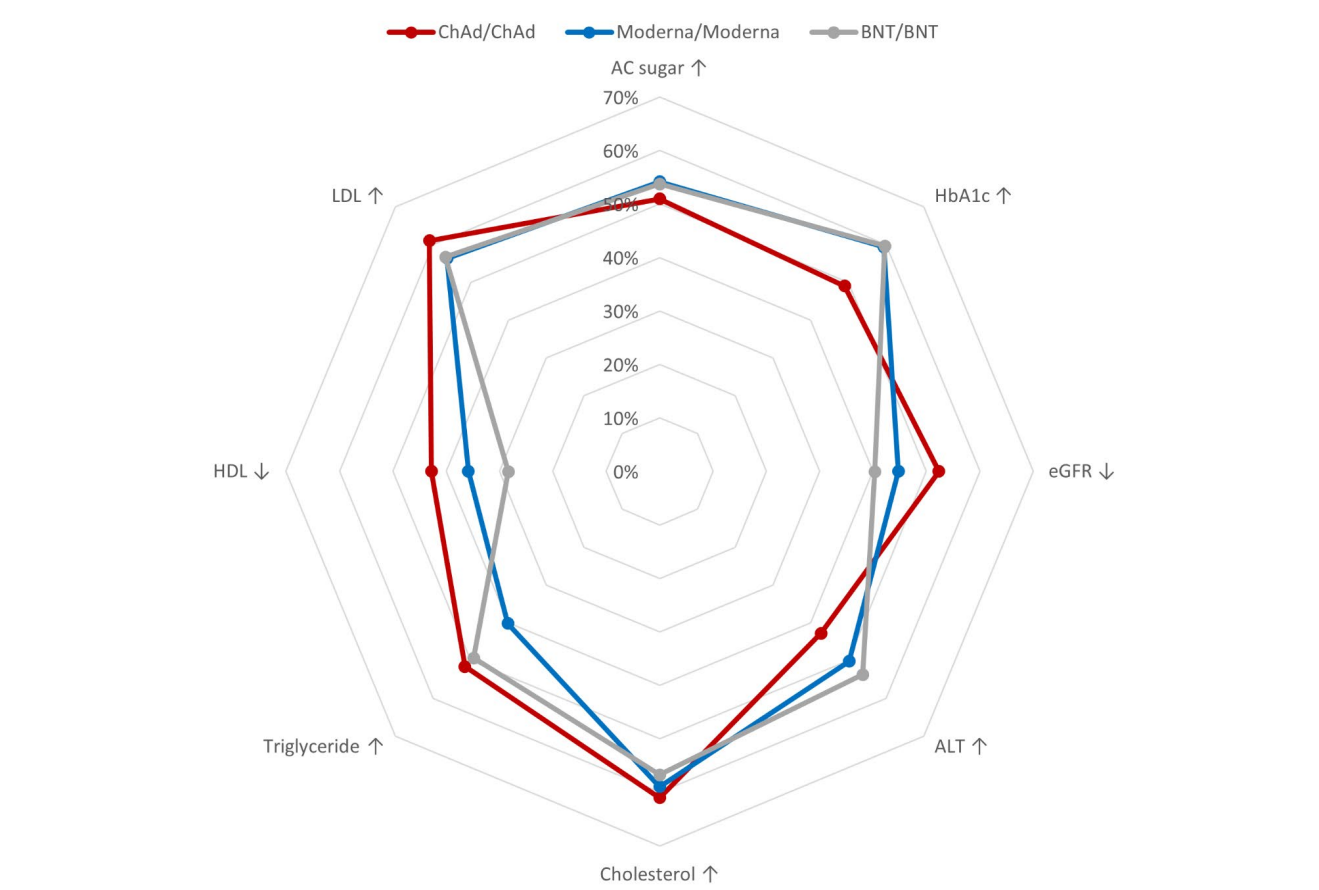
Percentage of subjects with unfavorable change in each biochemical parameter among participants following COVID-19 vaccination **Figure legend**: Moderna and BNT groups had around 60% of patients with elevated HbA1c following two doses of COVID-19 vaccination, while the ChAd group had only 49%

Additionally, we described the percentage of subjects with unfavorable changes in each biochemical parameter in each vaccine group by radar picture (Fig. 1). Concerning glycemic change, both Moderna and BNT groups had around 60% (59.39% and 59.7 respectively) of patients with elevated HbA1c following two doses of COVID-19 vaccination, and the ChAd group had only 49%. Elevation of cholesterol was seen in 61% of subjects with ChAd, 59% in Moderna and 57% in BNT, as well as the percentage of elevated low-density lipoprotein.

### Impacts on the elevated HbA1c

**Fig. 2:**
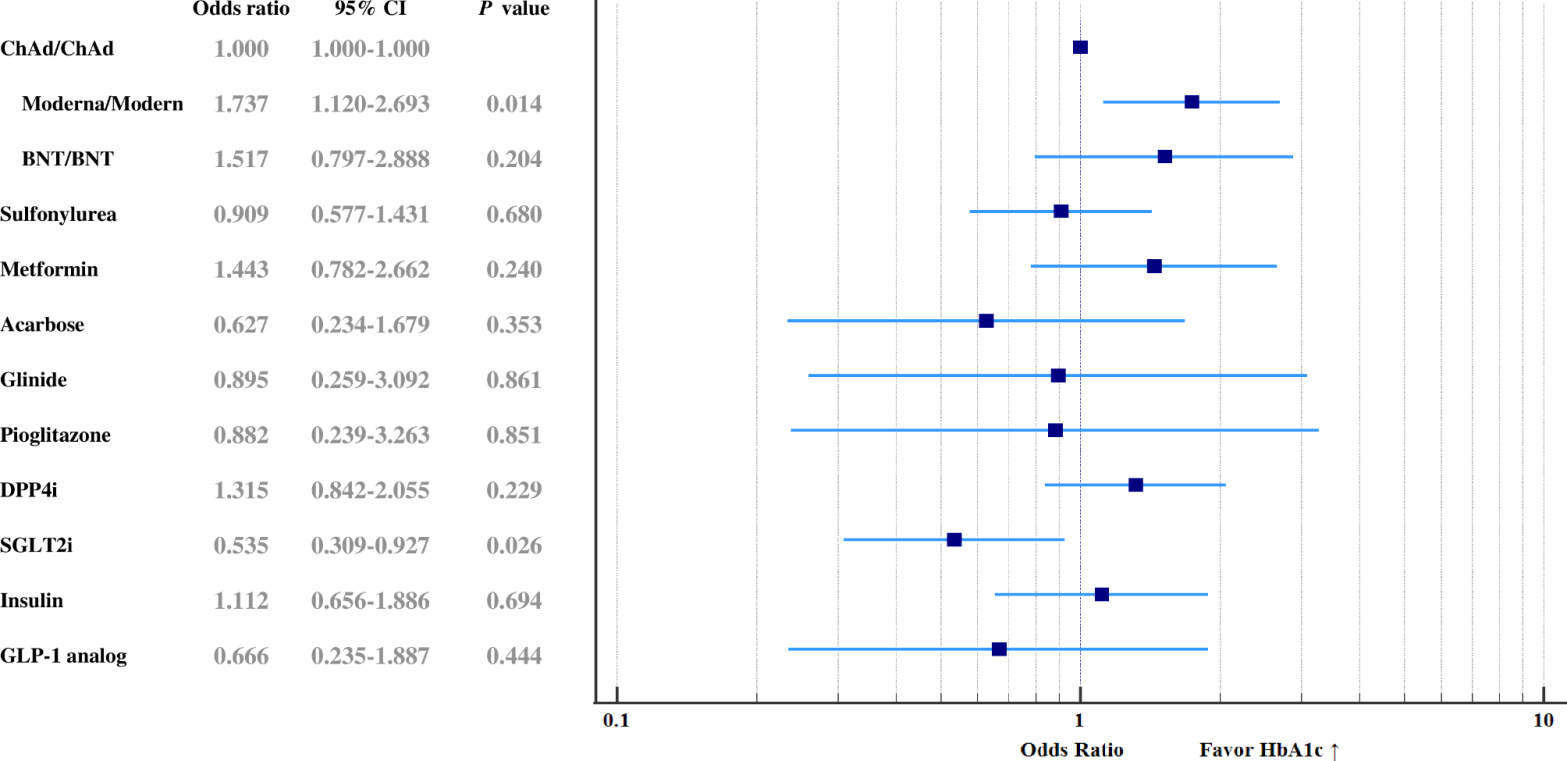
Forest plot of the effects of each COVID-19 vaccine and each anti-diabetic drug on the elevation of HbA1c among participants, determined using logistic regression modeling **Figure legend**: Moderna vaccines independently predict the elevation of HbA1c (OR 1.737, 95% CI 1.12–2.693, *P* = 0.014), and SGLT2i was negatively associated with it (OR 0.535, 95% CI 0.309–0.927, *P* = 0.026)

Figure 2 shows a forest plot of the effects of each kind of vaccine and each anti-diabetes drug on the post-vaccination-elevated HbA1c under adjusted co-variables, established using logistic regression modeling. Among the confounding factors, age was independently associated with elevated HbA1c (odds ratio 0.977, 95% confidence interval 0.958─0.996, *P* = 0.021) in the current multivariate logistic regression model. After adjustment for age and other confounding factors and based on the ChAd vaccine as a reference, the Moderna vaccine was found to independently predict the elevation of HbA1c with an odds ratio of 1.737 and a 95% confidence interval of 1.12 to 2.693, while the odds ratio in the BNT vaccine was 1.517 with 95% CI: 0.797 to 2.888.

Notably, among anti-diabetes drugs, the SGLT2i was found to be negatively associated with elevated HbA1c in patients following two doses of COVID-19 vaccination (OR 0.535, 95% CI 0.309–0.927, *P* = 0.026).

## Discussion

This study disclosed the glycemic change following two doses of various COVID-19 vaccinations. The increased average value of HbA1c (7.09–7.34%) was significantly noted in subjects receiving two doses of BNT vaccines, while both the other two groups of subjects taking Moderna or ChAd vaccines had mild increases in HbA1c without statistical significance. Regarding the percentage of subjects having elevated HbA1c, the patients receiving BNT and Moderna vaccines shared the same higher rates (around 60%) in comparison with the subjects with ChAd vaccines (49%), and the regression model revealed that the Moderna vaccine independently predicted subsequent increase in HbA1c. These findings suggested that patients with diabetes who received mRNA vaccines had higher possibility of stimulating the raising of serum glucose than the adenoviral vector vaccines. Several cases series have reported the hyperglycemia events post-COVID-19 vaccination, with two case series recording a total of six cases presenting with post-vaccine hyperglycemia following the first dose of ChAd vaccine [8, 10], while similar hyperglycemia conditions have also been reported following the first dose of mRNA vaccine in one case series (one with BNT and two with Moderna) and one case report with BNT vaccine [7, 9]. Accordingly, vaccination could be followed by mild to moderate elevation of serum glucose levels, and our current study further disclosed the influence on glucose metabolism following two doses of COVID-19 vaccination.

Several hypotheses have been proposed to explain how SARS-CoV-2 exacerbates hyperglycemia including damage of pancreatic beta cells [15, 16], the effects of acute stress-related hormones such as cortisol and catecholamines [17] or immunological dysregulation triggered by the infection that leads to a systemic cytokine response [18–20]; nevertheless, regarding possible cause of vaccine-associated hyperglycemia, no exact mechanism has been reported. It has been reported that post-vaccination hyperglycemia might be caused by pancreatic injury or pancreatitis in individuals who had received a COVID-19 vaccine [21], but this might not be relevant to our study since no pancreatitis event was observed. A more reasonable explanation for its occurrence is stimulation of the immune system and sympathoadrenal system, which leads to a physiological stress response and subsequent increased proinflammatory cytokines like tumor necrosis factor-α, or interleukin-1 and − 6, and counterregulatory hormones like cortisol and catecholamine [17]. Consequently, insulin sensitivity and glucose metabolism could change, resulting in glycemic instability. Notably, post-vaccination hyperglycemia has also been reported in cases with influenza vaccination [22], which suggests not just a reaction to the attenuated virus but to the vaccine-related excipients as well. The adjuvants used in vaccines for purpose of immunogenicity enhancement might trigger undesired inflammation reactions. Interestingly, the mRNA vaccines were reported to exhibit stronger immune response than adenoviral vector vaccines, either in humoral immune response or adverse discomfort [23–25], and these stronger responses were suspected of accompanying more stress response and therefore higher possibility of stress hyperglycemia, explaining the current finding of higher hyperglycemia possibility in our subjects receiving mRNA vaccines. One study in Europe using a spontaneous reporting system also presented mRNA COVID-19 vaccines as being associated with an increased reporting frequency of hyperglycemia events compared to adenoviral vector vaccines [26].

Furthermore, our current study suggested the use of SGLT2i as an anti-diabetes drug would reduce the possibility of deteriorated glycemic control regardless of the type of COVID-19 vaccine. The other anti-diabetes drugs didn’t have such significant impacts on maintaining glucose stability, even the insulin or GLP-1 analog. It was noted that SGLT2i was also associated with a lower mortality rate in individuals with diabetes suffering from COVID-19 [27, 28]. Since cytokine storm and its associated syndrome is linked to organ failure and mortality in patients with COVID-19 [19], the clinical benefit from SGLT2i was speculated to relate to its anti-inflammatory activity. Beyond the hypoglycemic function, SGLT2i was proven to possess numerous pleiotropic effects, particularly on a decrease in inflammatory cytokines such as tumor necrosis factor-αor interleukin-6, etc., as well as reduction of oxidative stress [29, 30]. As mentioned above, post-vaccination hyperglycemia was also speculated to associate with immune response and subsequent inflammation reaction, so SGLT2i had theoretical advantage on glycemic stability for patients receiving the COVID-19 vaccine over other anti-diabetes drugs.

The other interesting findings in our study were the subtly elevated eGFR and cholesterol/LDL in patients following COVID-19 vaccination. A comprehensive study on the pathophysiological alterations in subjects receiving COVID-19 vaccines revealed similar findings in lipids but diverse results in renal function [6]. Owing to lack of research evidence, the specific pathophysiologic link and causality between the biochemistry change and COVID-19 vaccination are difficult to confirm; nevertheless, we speculated that the elevated eGFR might be related to concordant hyperglycemia leading to the consequence of glomerular hyperfiltration [31], as that was also relatively obvious in subjects with mRNA vaccines. Since the higher percentage of subjects taking SGLT2i in the BNT vaccine group, the elevated eGFR (89.23 to 93.24 ml/min/1.73m2, *P* = 0.03) might have been attributed to the SLGT2i-associated renal protection [32–34]. The inflammation reaction post-vaccination might also contribute to the change of glomerular filtration rate and lipid metabolism in addition to the impact on serum glucose [35, 36]. Additionally, most of these patients were taking statins (around 70%), which might have prevented notable changes in their lipid profiles.

The present study carries some limitations because of its retrospective design. As a result of government policy in vaccine distribution, the BNT vaccine was initially reserved for younger populations and therefore less subjects being administered BNT vaccines were enrolled. Additionally, exercise habit and dietary behavior would also influence glycemic control, and these parameters were not available in this study. Further prospective precise studies are warranted to verify causality; nevertheless, we consider accession to and analysis of these metabolic changes among patients with diabetes following COVID-19 vaccine is valuable.

## Conclusions

In conclusion, patients with diabetes might have subtly increased glycemic status following two doses of COVID-19 vaccination, particularly with mRNA vaccines. Clinicians should be aware of glycemic perturbation possibility for such patients following vaccination. SGLT2i showed some protective effect on glycemic stability rather than other anti-diabetes drugs for patients receiving vaccines; nevertheless, vaccine hesitancy in patients with diabetes was not recommended with respect to the manageable metabolic changes.

## Data Availability

All data generated or analyzed during this study are included in this published article.

## List of abbreviations

COVID-19: coronavirus disease 2019
SARS-CoV-2: severe acute respiratory syndrome coronavirus 2
ChAd: ChAdOx1 nCov-19 (Oxford–AstraZeneca)
BNT: BNT162b2 (Pfizer–BioNtech)
DPP4i: dipeptidyl peptidase-4 inhibitor
SGLT2i: sodium-glucose co-transporter 2 inhibitor
GLP-1: glucagon-like peptide 1
AC: pre-meal
HbA1c: glycated hemoglobin
ALT: alanine aminotransferase
eGFR: estimated glomerular filtration rate
HDL: high density lipoprotein
LDL: low density lipoprotein
OR: odds ratio
CI: confidence interval
